# Mapping the Landscape of Do-it-Yourself Medicine

**DOI:** 10.5334/cstp.553

**Published:** 2022-12-15

**Authors:** ANNA WEXLER

**Affiliations:** Department of Medical Ethics and Health Policy, Perelman School of Medicine, University of Pennsylvania, 423 Guardian Drive, Philadelphia, PA 19104, US

**Keywords:** do-it-yourself, DIY medicine, biomedical citizen science, neurostimulation, fecal microbiota transplant, artificial pancreas

## Abstract

The practice of medicine is typically conceptualized as remaining within the boundaries of a hospital or clinic. However, in recent years, patients have been able to gain access to information about medical research as it is ongoing. As a result, there has been a rise in do-it-yourself (DIY) medicine, where individuals treat themselves for medical conditions outside of clinical settings, often mimicking experimental therapies that remain inaccessible to the wider public. For example, in DIY brain stimulation, individuals suffering from depression build at-home electrical headsets using nine-volt batteries, mimicking an experimental neuroscience technique used in scientific laboratories. In DIY fecal transplantation, those with intestinal disorders like *C. Difficile* and inflammatory bowel disease transplant stool from donors into themselves with the aid of blenders and enemas. In the open Artificial Pancreas System movement, diabetes patients hacked together an artificial pancreas system from their glucose monitors and insulin pumps, years before such a system was approved by the United States Food and Drug Administration (US FDA). To date, scholarship on DIY medicine has largely been relegated to specific medical domains (e.g., neurology, gastroenterology, infectious disease). In this paper, however, I recognize DIY medicine as a cross-cutting phenomenon that has emerged independently across medical domains but shares common features. I map the varieties of DIY medicine across these domains and suggest that four key factors lead to their creation, growth, and uptake. In doing so, this essay sheds light on an understudied area of biomedical citizen science that is likely to grow substantially in the coming decades.

## INTRODUCTION

Citizen science encompasses a wide range of scientific activities that involve participation from the general public (Eitzel et al. 2016; Haklay et al. 2021; Hecker et al. 2019). While many citizen science endeavors are related to environmental and ecological sciences (Kullenberg and Kasperowski 2016), a subset are specifically related to health and biomedical research. These include lay individuals conducting biological research, individuals contributing health data in novel ways to scientific projects, and increased patient engagement in biomedical research, among others (Guerrini et al. 2019; Trejo et al. 2020; Wiggins and Wilbanks 2019).

This essay takes as its focus one particular form of biomedical citizen science: patients who have endeavored to advance therapeutics for their own condition, often replicating experimental, but as-yet unavailable, therapies in their own homes. For example, in what has become known as “do-it-yourself” (DIY) brain stimulation, individuals suffering from depression build at-home electrical headsets using nine-volt batteries, mimicking an experimental neuroscience technique used in scientific laboratories (Jwa 2015; Wexler 2016). In DIY fecal transplantation, those with intestinal disorders like *C. Difficile* source stool from donors and transplant it into themselves with the aid of blenders and enemas, replicating novel clinical research (Ekekezie et al. 2020). In DIY hormone replacement therapy, transgender individuals source and self-administer hormones outside the physician’s office (de Haan et al. 2015; Rotondi et al. 2013). In DIY diabetes movements, patients have hacked together an artificial pancreas from their glucose monitors and insulin pumps (Burnside et al. 2020; Jennings and Hussain 2020). The rise of these DIY medical movements has been fueled by the internet, which has laid the foundation for online health communities and has allowed patients to gain greater access to information about medical research as it is ongoing.

To date, scholarship on DIY medical endeavors has largely been relegated to discussion within medical specialties (e.g., neurology, infectious disease, endocrinology). In this essay, however, I conceptualize DIY medicine as a cross-cutting social phenomenon that has emerged independently across multiple medical domains, yet shares common features. First, I provide background on DIY medicine and how the term has been used contemporarily. Second, I describe several case studies of DIY medical movements in which scholars have documented loose-knit communities of individuals coming together to innovate, replicate, or treat themselves outside of clinical settings. Third, I suggest that four key factors set the stage for any DIY medical movement: frustrated patients, lack of access to an effective treatment, online social media outlets, and the availability of a therapeutic that is relatively easy to create or access. Finally, I conclude by discussing the outlook for the future of DIY medicine.

## WHAT IS DO-IT-YOURSELF MEDICINE?

In citizen science, the term “citizen” modifies the word “science” to reflect that *who* is doing the science represents a departure from societal norms: it is lay individuals, not trained scientific professionals. Similarly, in do-it-yourself medicine, the term “do-it-yourself” signals that lay individuals, not medical experts, are practicing medicine. This modifier (DIY) is therefore laden with meaning, conveying both what the societal norm is regarding the expertise required for an activity as well as how a specified practice departs from it. For example, we would assume that one speaking of doing a “DIY bathroom remodel” was not a professional contractor, but rather a layperson carrying out a remodeling project, perhaps with the aid of YouTube videos. But a professional contractor—with expertise in home remodeling—would not speak of a “DIY bathroom model” as the practice would not be DIY. By the same token, it would be paradoxical to use DIY in reference to activities that are not typically viewed as requiring expertise, such as “DIY housework” or “DIY laundry.”

The term “do-it-yourself” has its roots in several movements across the past century (Ferretti 2019). Cultural studies scholar Grewe-Salfeld (2021) has pointed to three “intersecting waves” of DIY, including a trend toward home improvement in the 1950s and 1960s, its usage in punk counterculture in the 1980s and 1990s, and the contemporary Maker Movement, which prioritizes creative physical activities. Some citizen science activities, particularly in the realm of biology, have been referred to as “DIY science” as they combine an ethos of open science, the culture of the Maker Movement, critiques of mainstream science, and a hacker culture (Delfanti 2013; Landrain et al. 2013).

While DIY medicine shares commonalities with the above movements—particularly in its existence outside of the mainstream—it also has its own distinct history. Centuries ago, medicine was not exclusively in the realm of the physician: it was viewed as common sense knowledge passed down through families (Rosenberg 2007; Starr 1982). In rural America in the eighteenth and nineteenth centuries, there was an emphasis on self-reliance for medical needs. Home medical guides, such as John Tennant’s *Every Man His Own Doctor* (1736), William Buchan’s *Domestic Medicine* (1771), and *Gunn’s Domestic Medicine* (1830), offered detailed instructions for the lay individual on how to treat various diseases. These books and other publications providing at-home medical advice have been referred to as DIY medicine, to reflect the practice of medicine in the absence of a trained physician (King 1967; Tomes 2016).

In contemporary usage, the term DIY has been applied in an ad-hoc manner to medical practices by journalists, scientists, clinicians, academic scholars, and even users themselves. Medical endeavors that have been described as DIY have included efforts to 3D print prosthetic limbs (Manero et al. 2019; Parry-Hill 2019); the practice of dentistry and orthodontics outside of a healthcare provider’s office (Carter and Stokes 2021; Westgarth 2021); the creation of low-cost hearing aids and user modifications to existing hearing loss technology (Brewster et al. 2019; Lesté-Lasserre 2020); an open-source version of an epinephrine auto-injector called the “Epi-Pencil” (Condliffe 2016); the release of instructions for synthesizing abortion pills (Koebler 2022); homemade COVID-19 healthcare supplies (Richterich 2020); COVID-19 vaccines developed outside of clinical trials (Caplan and Bateman-House 2020); and efforts to develop open-source versions of insulin (Gallegos and Peccoud 2018), among others.

DIY medicine is most commonly used in reference to the practice of medicine that exists outside the mainstream, mostly with regard to who is doing it (i.e., those without professional medical training), but occasionally also regarding how it is being done (i.e., outside of typical medical or scientific clinical or research settings). For example, a BBC article about surgeons operating in remote settings using improvised surgical tools referred to the practice as “DIY medicine,” even though the individuals described were all trained medical professionals (Gorvett 2016). When the term DIY is used by the media in reference to medical techniques, there is often a sensational appeal. In contrast, professional scientists, clinicians, and scholars sometimes use the term to delineate boundaries between the “correct” practice of medicine and DIY practices that may be “unsafe” or “dangerous” (ADA 2018; Wurzman et al. 2016). Along these lines, even practices that involve consumers purchasing products from companies, such as direct-to-consumer genetic and medical tests, have sometimes been described by clinicians and scholars as DIY (Borry, Sénécal, and Knoppers 2016; McClurg 2018).

Although many of the abovementioned applications of the term DIY to medical practice are one-off usages, typically describing an isolated case, this essay takes as its focus instances of DIY medicine that represent more significant and sustained social phenomena, in which loose-knit communities of patients work to replicate or advance therapeutics for their own condition. The examples mentioned in the Introduction—of DIY neurostimulation, DIY fecal transplants, DIY hormone replace therapy, and the DIY artificial pancreas system—are all cases where the term DIY has been used consistently over time by the media, scholars, and even users themselves to describe their practices.

These DIY medical endeavors differ from other kinds of biomedical citizen science in several ways. First, unlike other initiatives that aim to increase patient engagement in traditional scientific knowledge production, these DIY medical endeavors are entirely disconnected from mainstream or establishment research. Second, those involved in DIY medicine are usually patients (or their caregivers) and therefore have a strong personal motivation to improve their own quality of life; this sets them apart from biomedical citizen science endeavors like DIY biology, in which participants are motivated by interests in open science and educational experimentation (Roosth 2017; Wexler 2017b). Third, in these cases of DIY medicine, participants use their own bodies as sites of experimentation, unlike other biomedical citizen science that involves the contribution of health data or research time. Fourth, while DIY medical movements sometimes emerge from online patient communities, they move beyond mere discussion groups in that they are oriented toward developing and utilizing a therapeutic intervention.

Multiple instances of this kind of DIY medicine have arisen independently across multiple medical domains, yet appear to share common features. The remainder of this essay therefore focuses exclusively on these DIY medical movements, which move beyond single cases of DIY medicine and represent the sustained efforts of a group over time.

## EXAMPLES OF DIY MEDICAL MOVEMENTS

This section describes four case studies of DIY medical movements in which there exists significant independent documentation (by scholars and journalists) of a loose-knit community of individuals coming together to innovate, replicate, or treat themselves outside of clinical settings, often mimicking advanced experimental therapies that remain inaccessible to the general public. The case studies described here are not intended to be a comprehensive representation of the entirety of DIY medical movements. Indeed, other endeavors may be considered DIY medicine, such as amyotrophic lateral sclerosis (ALS) patients self-administering experimental treatments (Wicks et al. 2011) and cluster headache patients developing and innovating psychedelic therapeutics (Kempner and Bailey 2019). However, the DIY movements depicted here (and summarized in Table 1) have all been consistently referred to as DIY medicine by independent observers and users themselves, and even the brief sketch of their features provided here reveals notable commonalities.

### DIY NEUROSTIMULATION

The DIY neurostimulation movement arose in the early 2010s, spurred by lay individuals reading media reports of early scientific success in the use of low levels of electrical brain stimulation for clinical indications (e.g., depression, anxiety, attention deficit disorder) as well as for cognitive enhancement (Jwa 2015; Wexler 2016). Multiple websites, fora, and YouTube videos arose to guide individuals on how to construct neurostimulation devices at home using parts sourced from local electronics stores (Wexler 2015). At-home neurostimulation was widely criticized by neuroscientists for being an “unorthodox” use of neurotechnology (Bikson, Bestmann, and Edwards 2013) and drew attention from government regulators and professional medical organizations (Wexler 2017a). Today, it is more common for individuals to purchase ready-made neurostimulation devices that are marketed for cognitive enhancement or wellness, even though users may utilize such devices for medical indications (Wexler 2016). While there has been occasional interest in crowdsourcing the data from DIY users of neurostimulation to advance scientific knowledge (Wexler and Hamilton 2017), it does not appear that any organized data-gathering efforts have taken place. The DIY neurostimulation movement never gained widespread public appeal, as some ethicists feared it might, but today it remains a subculture with a moderately active Reddit forum (Wexler 2017b).

### DIY FECAL MICROBIOTA TRANSPLANTS (FMT)

DIY fecal microbiota transplants (FMT) emerged in 2010 when patients learned of the promising results being reported in the scientific literature for using stool transplants to treat resistant *Clostridium difficile* (*C. Diff*) infections (Eakin 2014). Rather than waiting for this treatment to become approved by the United States Food and Drug Administration (US FDA), those suffering from recurrent *C. Diff* infections began to conduct stool transplants at home, sourcing stool donations from family members, friends, and strangers (Kremer 2014). They learned about the practice through how-to websites, blogs, and videos (Ekekezie et al. 2020). In 2013, FMT became more widely available in clinical settings in the US after the FDA stated that it would exercise enforcement discretion (i.e., not enforce existing regulations) for FMT when used for treatment-resistant *C. Diff* infections (Khoruts, Hoffmann, and Palumbo 2019). Today, because FMT for treatment-resistant *C. Diff* has become more accessible, the DIY FMT movement has shifted to involve individuals using the technique for experimental indications beyond *C. Diff* (Ekekezie et al. 2020). Physicians have continuously warned against DIY FMT due to the possibility of unintentionally self-transplanting harmful microbes, such as HIV or hepatitis, through donor stool. Even in clinical settings, where donor stool is screened, there can still be risks: in 2019, a patient in a clinical trial for FMT died and another became seriously ill after contracting a drug-resistant strain of *E. Coli* from donor stool (DeFilipp et al. 2019; FDA 2019).

### DIY OPEN ARTIFICIAL PANCREAS SYSTEM (APS)

In 2014, a number of patients and caregivers affected by type 1 diabetes created a closed-loop system that uses an algorithm to pair their continuous glucose monitors and insulin pumps (Lewis 2019). In this system, insulin delivery is automated and left entirely to the algorithms (Lewis, Leibrand, and #OpenAPS Community 2016; Omer 2016). Blueprints for the system—which came to be known as the Open Artificial Pancreas System (OpenAPS) because its algorithms mimic the insulin delivery of a normally functioning pancreas—were published online and gave rise to an online community of users (Jennings and Hussain 2020). Compared with other DIY medical movements, the DIY Open APS movement is highly organized—its users contribute, collect, and analyze their own data, and publish articles in academic journals. Individuals using DIY systems have continuously reported improvements in their quality of life (Asarani et al. 2020; Braune et al. 2019; Litchman et al. 2019; Palmer et al. 2020). However, professional groups have issued statements advising healthcare providers against recommending DIY diabetes systems (Jennings and Hussain 2020), and the FDA has warned against the use of unauthorized devices for diabetes management after a patient suffered an accidental insulin overdose (Cortez 2019). Although commercial automated insulin delivery systems are gradually becoming available in some countries, some users still may opt for a DIY system because of its lower cost or a lack of local availability of a commercial system (Lewis and Hussain 2022).

### DIY HORMONE REPLACEMENT THERAPY (HRT)

Although the use of black-market hormones among transgender persons dates back at least twenty years (Clements-Nolle et al. 2001), scholarship documenting the phenomena that has come to be known as DIY HRT was published in the early 2010s (de Haan et al. 2015; Rotondi et al. 2013). There are currently active fora and websites related to DIY HRT that advise users on dosage, transition protocols, and outlets for sourcing hormones (Branstetter 2016; Santora 2020). Studies conducted in Canada (Rotondi et al. 2013), the US (de Haan et al. 2015; Sanchez, Sanchez and Danoff 2009), the United Kingdom (Mepham et al. 2014), Brazil (Maschião et al. 2020) and Sweden (Fondén 2020) have all described similar phenomena of DIY or non-prescribed hormone use, finding that anywhere from twenty to eighty percent of all individuals surveyed have sourced hormones outside the auspices of the physician’s office. One study found the practice to be more prevalent in trans women compared with trans men (Mepham et al. 2014). Reasons for turning to DIY HRT include lack of access to specialized care due to stigma and discrimination (Metastasio et al. 2018), past negative experiences with providers and limited financial resources (Rotondi et al. 2013), and not being able to see a provider (de Haan et al. 2015). Unlike the widespread controversy garnered by the DIY medical movements described above, physicians and professional medical societies have not spoken out widely about DIY HRT, although at least one paper has advocated for increasing awareness among clinicians of the phenomenon (Metastasio et al. 2018).

## KEY FACTORS FOR DIY MEDICAL MOVEMENTS

Understanding the social contexts that give rise to DIY medical movements can help predict where and how they may arise in the future. Here, I suggest that four key factors set the stage for the emergence of a DIY medical movement. First, there is typically a population of individuals who are suffering from a disease, disorder, or condition. These individuals are often frustrated or dissatisfied with their existing care or lack of effective therapeutics, or the pace at which an experimental therapy may trickle down to them; others are desperate for relief from their conditions. They therefore have a strong intrinsic motivation to seek out a therapeutic for themselves or their loved ones.

The second key factor is an ability to rapidly communicate information: As noted in the Introduction, the internet has provided a means of accessing information about medical research, as well as allowed for the development of social media and online fora that have fostered the growth of patient communities. Indeed, the DIY medical movements described above have all centered on online patient fora—typically on Facebook or Reddit—where users can quickly share and exchange information. The ability for rapid, multi-directional information exchange sets contemporary DIY medical movements apart from the one-way publication of medical information offered by home medical books and magazines of previous centuries (Greene 2016; King 1967).

The third key factor is that individuals lack access to a given therapy or procedure. Access barriers can take different forms: a therapy or innovation may be experimental (i.e., being studied in laboratory settings) and as-yet unapproved, such as in the case of DIY brain stimulation; the technology may also not exist, such as in the case of the closed-loop artificial pancreas systems. Even if a therapy is approved, individuals may be unable to access it due to cost or lack of local availability, such as in DIY HRT and DIY FMT. Other reasons may include hesitancy to access care, such as in DIY HRT, where transitioning individuals may fear discrimination or may have had prior negative experiences with healthcare providers.

Fourth, for a DIY movement to gain traction, the therapeutic should be relatively easy for individuals to access or create. For example, in DIY neurostimulation, users were able to build at-home electrical brain stimulation devices using simple parts sourced from electronics stores. However, the home use of other kinds of neurostimulation—such as transcranial magnetic stimulation—never took off, likely because their construction is far more complex. DIY FMT involves sourcing stool, a material that anyone can access, and online pharmacies readily provide hormones to those interested in DIY HRT. It is likely that the greater the accessibility or replicability of a therapeutic, the more widely a DIY medical movement may spread.

Other characteristics of DIY medical movements are not essential features, but still bear mentioning. For example, there often arises an informal marketplace where purveyors sell supplies related to the DIY activity, such as neurostimulation devices and “brain device kits” offered by entrepreneurs in the realm of DIY neurostimulation (Wexler 2015); individuals selling their own stool in DIY FMT (Ma et al. 2017); and online pharmacies offering black-market hormones for those interested in DIY HRT (Branstetter 2016). In addition, DIY medical movements are often controversial as they raise challenges for clinicians, professional medical societies, and regulators, who struggle with how to navigate the home use and self-administration of therapies that have typically remained behind the closed doors of the medical clinic. Thus, the emergence of a DIY medical movement may be accompanied by publications advising against a DIY practice, such as commentaries and essays from individual physicians, position papers and statements from professional organizations, and warnings from government regulators.

Finally, it is worth noting that while DIY medicine may be an alternative practice that exists outside mainstream medicine, its users appear to have a strong affinity for science and medicine: One study of DIY neurostimulation found that users were “early adopters of technology with a penchant for reading scientific articles” (Wexler 2018), and a survey of users of automated insulin delivery systems found that 45% of respondents had a professional background in science or medicine (Braune et al. 2021). By contrast, users of complementary and alternative medicine (CAM) and those who are vaccine-hesitant tend to have a more negative view of mainstream medicine, fearing that conventional medical interventions may be harmful (Ernst 2001). While online surveys of users of DIY neurostimulation, DIY FMT, and DIY diabetes have found that users tend to be white, highly educated, and of higher socioeconomic status (Ekekezie et al. 2020; Wexler 2018), the population of users of DIY HRT does not appear to share these same demographic characteristics (de Haan et al. 2015; Sanchez, Sanchez, and Danoff 2009).

## THE FUTURE OF DIY MEDICAL MOVEMENTS

Positioning DIY medical movements within the framework of the four abovementioned factors can yield deeper knowledge of when the next DIY medical movement might occur. For example, the recent United States Supreme Court decision in *Dobbs v. Jackson,* which paved the way for states to reduce abortion access, portends a potential DIY abortion movement. Three key factors are apparent: frustrated and desperate patients, barriers to access, and the ability to exchange information on online fora. The fourth factor—an abortion method that is relatively easy to access or replicate—may already be present: a procedure known as menstrual extraction was developed in the 1970s and involves the use of a mason jar, a tube, and a syringe (Baker 2022; Chalker and Downer 1996; Marty 2019). The surge of interest on social media for menstrual extraction (Dawson 2022) may have the potential to spread into a DIY medical movement in locations where abortion is restricted and travel to other states may be difficult.

The COVID-19 pandemic also contained many of the key factors of a DIY medical movement: patients who were fearful of contracting disease, the ability to communicate information online, and access barriers (i.e., the lack of an effective therapeutic or preventative). Thus, when media reports emerged of a potentially beneficial therapeutic, hydroxychloroquine, that was relatively easy to access, individuals began to source and self-administer it, resulting in at least one death (Neuman 2020). Similarly, individuals fearful of contracting COVID-19 began to develop and test DIY COVID vaccines, and even published a guide on how to make vaccines at-home (Guerrini et al. 2020). However, DIY COVID-19 vaccines never gained widespread traction, likely due to the difficulty of creating the vaccine, lack of data indicating efficacy, and the development of a commercially available vaccine.

The above framework can also help conceptualize related phenomena that may be similar to DIY medical movements. For example, there are many online groups of patients who are frustrated by their quality of care and the lack of effective therapeutics for their condition; in many cases, however, drugs that are being tested in clinical trials for these conditions are synthesized exclusively by pharmaceutical companies and are unavailable to patients. The frustration at the lack of access to these drugs has resulted in the “right to try” movement, where patients and advocacy groups have campaigned for alternative pathways for earlier access to experimental pharmaceuticals (Folkers, Chapman and Redman 2019). However, the “right to try” movement differs from DIY medical movements because in the former case, a therapeutic is manufactured by a single company and is therefore not easy to access or replicate—it lacks the fourth key factor. Thus it is unlikely that DIY medical movements will arise for experimental therapeutics involving novel pharmaceutical drugs.

In theory, DIY medical movements should have the ability to advance scientific knowledge in some way. However, apart from the DIY diabetes movement, few have successfully contributed to science in a significant manner. Although further research is needed to examine the definitive reasons for this, here I offer several points for consideration. First, patients in DIY medical movements appear to be motivated by improving their own condition, not generating new scientific knowledge. Thus, if their DIY practices are successful—and there is evidence of high rates of satisfaction across DIY neurostimulation, DIY FMT, and DIY diabetes (Wexler 2018; Ekekezie et al. 2020; Palmer et al. 2020)—individuals may not be motivated to take added steps to gather, share, and analyze their own data. Second, there are difficulties in producing valid and reliable data when DIY practices vary considerably, such as in DIY neurostimulation and DIY FMT, where there are no standardized dosages or established methods of administration. Therefore, the ability to compile and compare data beyond anecdotal n-of-1 accounts may remain limited.

Third, a high level of group organization and leadership appears to be necessary to generate data that can be viewed as legitimate by mainstream science. The DIY diabetes movement has been a notable outlier in this respect, collecting, analyzing, and publishing their data in scientific journals. Fourth, for community-generated data to be published in academic journals, individuals involved in generating or analyzing the data need training in the norms of academic science writing—or they must partner with professional scientists. Here, too, the DIY diabetes movement has shown remarkable aptitude, forming collaborations with scientists and physicians, and publishing co-authored articles (e.g., Burnside et al. 2022). Further research to understand the success of the DIY diabetes movement may be instructive for other DIY medical endeavors.

Fifth, because healthcare providers and professional medical societies typically view DIY medicine as dangerous or unsafe, DIY medical movements may face significant barriers to making progress. Even physicians who may see the value in collecting crowdsourced data may be hesitant to get involved with DIY medical movements, either due to fears of being associated with a fringe movement or due to concerns about liability (i.e., in terms of providing medical advice to patients or assisting them with procuring supplies). Thus, unlike other areas of citizen science where scientists form partnerships with lay individuals, there is often a rift between physicians and participants in DIY medical movements.

## CONCLUSION

The four factors outlined in the paper—frustrated patients, online fora, access barriers, and the ease of creation or acquisition of the therapeutic—are all key factors that set the stage for the creation of a DIY medical movement. For clinical indications that lack an effective treatment, and for which a therapeutic device or procedure is relatively easy to create or access, it is conceivable that a DIY medical movement will emerge, almost certainly centered on an online social media outlet. As noted above, the chances of DIY medical movements forming around new drugs are low, due to the difficulty in accessing or synthesizing a novel pharmaceutical.

DIY medical movements will almost certainly continue to raise controversy, as the spread of information, particularly on the internet and via social media, cannot be readily restricted. Furthermore, government agencies such as the FDA regulate the sale of medical products across state lines—not what individuals do to their bodies in the privacy of their own homes. Additional research is therefore needed to study the most beneficial methods for clinicians, professional societies, and regulators to address the challenges raised by DIY medical movements.

Finally, DIY medical movements have shown promise for those who need them most—patients who are frustrated with the perceived inadequacy of healthcare and the barriers to accessing effective therapeutics. While some DIY medical movements have not made significant contributions to science, the DIY diabetes movement has been a notable outlier in terms of its level of organization, ability to gather and publish data, and to partner with scientists. Further research to understand the features that have led to its success may yield strategies for future DIY medical movements to become partners, rather than foes, with physicians and scientists.

## Figures and Tables

**Table 1:** Summary of the four do-it-yourself (DIY) medical movements described in this section.

DIY MEDICAL MOVEMENT	POPULATION	ACCESS BARRIER	DIY ACTIVITY	RELEVANT MEDICAL SPECIALTY
**DIY neurostimulation**	Individuals with depression, anxiety, and ADHD; healthy individuals	Technology still experimental	Building at-home neurostimulation devices and stimulating their brains with electricity	Neurology and Psychiatry
**DIY fecal microbiota transplant (FMT)**	Individuals suffering from recurrent intestinal infections	Technique not available outside of limited clinical trials	Procuring donor stool from family, friends, and/or online sellers, and transplanting into their colons	Infectious disease and Gastroenterology
**DIY diabetes movements**	Type 1 diabetes patients and their caregivers	Specific technology (automated insulin delivery system) did not exist	Hacking into their glucose monitors and insulin pumps to create automated insulin delivery systems	Endocrinology
**DIY hormone replacement therapy (HRT)**	Transgender individuals	Stigma; cost; lack of access to provider with relevant expertise	Sourcing and self-administering hormones	Endocrinology

## References

[R1] American Dental Association (ADA). 2018. ADA launches public awareness campaign discouraging DIY dentistry. 9 August 2018. Available at http://web.archive.org/web/20210516205325/https://www.ada.org/en/publications/ada-news/2018-archive/august/ada-launches-public-awareness-campaign-discouraging-diy-dentistry?nav=news. Accessed on September 1, 2022.

[R2] Asarani NAM, Reynolds AN, Elbalshy M, Burnside M, de Bock M, Lewis DM and Wheeler BJ. 2020. Efficacy, safety, and user experience of DIY or open-source artificial pancreas systems: a systematic review. Acta Diabetologica, 1–9. DOI: 10.1007/s00592-020-01623-4 33128136

[R3] Baker CN. 2022. Abortion how-to: The Ms. Q&A on menstrual extraction with Carol Downer. Ms. Magazine, July 14. Available at: https://msmagazine.com/2022/07/14/abortion-how-to-carol-downer-menstrual-extraction. Accessed on September 10, 2022.

[R4] Bikson M, Bestmann S and Edwards D 2013. Transcranial devices are not playthings. Nature, 501(7466): 167–167. DOI: 10.1038/501167b PMC4326099 24025832

[R5] Borry P, Sénécal K and Knoppers BM. 2016. Do-it-yourself newborn screening. JAMA Pediatrics, 170(6): 523. DOI: 10.1001/jamapediatrics.2016.0166 27111240

[R6] Branstetter G 2016. Sketchy pharmacies are selling hormones to transgender people. The Atlantic, 31 August. Available at: https://www.theatlantic.com/health/archive/2016/08/diy-hormone-replacement-therapy/498044. Accessed on September 1, 2022.

[R7] Braune K, Gajewska KA, Thieffry A, Lewis DM, Froment T, O’Donnell S, Speight J, Hendrieckx C, Schipp J, Skinner T, Langstrup H, Tappe A, Raile K and Cleal B 2021. Why #WeAreNotWaiting—motivations and self-reported outcomes among users of open-source automated insulin delivery systems: multinational survey. Journal of Medical Internet Research, 23(6): e25409. DOI: 10.2196/25409 34096874 PMC8218212

[R8] Braune K, O’Donnell S, Cleal B, Lewis D, Tappe A, Willaing I, Hauck B and Raile K 2019. Real-world use of do-it-yourself artificial pancreas systems in children and adolescents with Type 1 Diabetes: Online survey and analysis of self-reported clinical outcomes. JMIR mHealth and uHealth, 7(7): e14087. DOI: 10.2196/14087 31364599 PMC6691673

[R9] Brewster S, Fitzpatrick G, Cox A, Kostakos V, O’Kane AA, Aliomar A, Zheng R, Schulte B and Trombetta G 2019. Social, cultural and systematic frustrations motivating the formation of a DIY hearing loss hacking community. Proceedings of the 2019 CHI Conference on Human Factors in Computing Systems, 1–14. DOI: 10.1145/3290605.3300531

[R10] Burnside M, Crocket H, Mayo M, Pickering J, Tappe A and de Bock M 2020. Do-it-yourself automated insulin delivery: A leading example of the democratization of medicine. Journal of Diabetes Science and Technology, 14(5): 878–882. DOI: 10.1177/1932296819890623 31876179 PMC7753855

[R11] Burnside MJ, Lewis DM, Crocket HR, Meier RA, Williman JA, Sanders OJ, Jefferies CA, Faherty AM, Paul RG, Lever CS, Price SKJ, Frewen CM, Jones SD, Gunn TC, Lampey C, Wheeler BJ and de Bock MI. 2022. Open-source automated insulin delivery in Type 1 Diabetes. New England Journal of Medicine, 387(10): 869–881. DOI: 10.1056/NEJMoa2203913 36069869

[R12] Caplan AL and Bateman-House A 2020. The danger of DIY vaccines. Science, 369(6507): 1035–1035. DOI: 10.1126/science.abe4440 32855312

[R13] Carter A and Stokes S 2021. Availability of ‘do-it-yourself’ orthodontics in the United Kingdom. Journal of Orthodontics, 49(1): 83–88. DOI: 10.1177/14653125211021607 34096369 PMC8915217

[R14] Chalker R and Downer C 1996. The Woman’s Book of Choices: Abortion, Menstrual Extraction, RU-486. New York City: Seven Stories Press.

[R15] Clements-Nolle K, Marx R, Guzman R and Katz M 2001. HIV prevalence, risk behaviors, health care use, and mental health status of transgender persons: implications for public health intervention. American Journal of Public Health, 91(6): 915–921. DOI: 10.2105/AJPH.91.6.915 11392934 PMC1446468

[R16] Condliffe J 2016. It costs $30 to make a DIY EpiPen, and here’s the proof. MIT Technology Review, 20 September. Available at: https://www.technologyreview.com/2016/09/20/157437/it-costs-30-to-make-a-diy-epipen-and-heres-the-proof. Accessed on September 13, 2022.

[R17] Cortez MF. 2019. Patient hurt by do-it-yourself artificial pancreas prompts FDA warning. Bloomberg, 17 May. Available at: https://www.bloomberg.com/news/articles/2019-05-17/patient-hurt-by-do-it-yourself-pancreas-prompting-fda-warning. Accessed on September 13, 2022.

[R18] Dawson B 2022. The underground abortion alternative being reconsidered after the Roe Leak. Mel Magazine, May 15. Available at: https://melmagazine.com/en-us/story/menstruation-extraction-roe-v-wade-leak. Accessed on September 14, 2022.

[R19] DeFilipp Z, Bloom PP, Soto MT, Mansour MK, Sater MRA, Huntley MH, Turbett S, Chung RT, Chen Y-B and Hohmann EL. 2019. Drug-resistant E. coli bacteremia transmitted by fecal microbiota transplant. New England Journal of Medicine, 381(21): 2043–2050. DOI: 10.1056/NEJMoa1910437 31665575

[R20] de Haan G, Santos G-M, Arayasirikul S and Raymond HF. 2015. Non-prescribed hormone use and barriers to care for transgender women in San Francisco. LGBT Health, 2(4): 313–323. DOI: 10.1089/lgbt.2014.0128 26788772

[R21] Delfanti A 2013. Biohackers: The Politics of Open Science. London: Pluto Press.

[R22] Eakin E 2014. The excrement experiment. 1 December 2014. Available at https://www.newyorker.com/magazine/2014/12/01/excrement-experiment. Accessed on September 8, 2022.

[R23] Eitzel MV, Cappadonna JL, Santos-Lang C, Duerr RE, Virapongse A, West SE, Kyba CCM, Bowser A, Cooper CB, Sforzi A, Metcalfe AN, Harris ES, Thiel M, Haklay M, Ponciano L, Roche J, Ceccaroni L, Shilling FM, Dörler D, Heigl F, Kiessling T, Davis BY and Jiang Q 2016. Citizen science terminology matters: Exploring key terms. Citizen Science: Theory and Practice, 2(1): 1. DOI: 10.5334/cstp.96

[R24] Ekekezie C, Perler BK, Wexler A, Duff C, Lillis CJ and Kelly CR. 2020. Understanding the scope of do-it-yourself fecal microbiota transplant. The American Journal of Gastroenterology, 115(4): 1. DOI: 10.14309/ajg.0000000000000499 31972620 PMC7359198

[R25] Ernst E 2001. Rise in popularity of complementary and alternative medicine: reasons and consequences for vaccination. Vaccine, 20: S90–S93. DOI: 10.1016/S0264-410X(01)00290-0 11587822

[R26] FDA. 2019. Important safety alert regarding use of fecal microbiota for transplantation and risk of serious adverse reactions due to transmission of multi-drug resistant organisms. 13 June 2019. Available at https://www.fda.gov/vaccines-blood-biologics/safety-availability-biologics/important-safety-alert-regarding-use-fecal-microbiota-transplantation-and-risk-serious-adverse. Accessed on September 9, 2022.

[R27] Ferretti F 2019. Mapping do-it-yourself science. Life Sciences, Society and Policy, 15(1): 1. DOI: 10.1186/s40504-018-0090-1 30741364 PMC6369552

[R28] Folkers K, Chapman C and Redman B 2019. Federal right to try: Where is it going? Hastings Center Report, 49(2): 26–36. DOI: 10.1002/hast.990 30998281

[R29] Fondén E 2020. Do it yourself hormone replacement therapy. Thesis; Lund University. Available at: https://lup.lub.lu.se/student-papers/search/publication/9027210. Accessed on September 8, 2022.

[R30] Gallegos JE and Peccoud J 2018. In the face of high costs, DIYers hope to brew their own insulin. Discover, 13 September. Available at: https://www.discovermagazine.com/health/in-the-face-of-high-costs-diyers-hope-to-brew-their-own-insulin. Accessed on September 3, 2022.

[R31] Gorvett Z 2016. DIY medicine at the edges of civilisation. BBC, 16 November. Available at: https://www.bbc.com/future/article/20161116-diy-medicine-at-the-edges-of-civilisation. Accessed on September 15, 2022.

[R32] Greene JA. 2016. Do-it-yourself medical devices — technology and empowerment in American health Ccre. New England Journal of Medicine, 374(4): 305–308. DOI: 10.1056/NEJMp1511363 26816009

[R33] Grewe-Salfeld M 2021. Biohacking, Bodies and Do-It-Yourself: The Cultural Politics of Hacking Life Itself. Bielefeld: transcript Verlag. DOI: 10.1515/9783839460047

[R34] Guerrini CJ, Sherkow JS, Meyer MN and Zettler PJ. 2020. Self-experimentation, ethics, and regulation of vaccines. Science, 369(6511): 1570–1572. DOI: 10.1126/science.abe1963 32943452 PMC8095856

[R35] Guerrini CJ, Wexler A, Zettler PJ and McGuire AL. 2019. Biomedical citizen science or something else? Reflections on terms and definitions. The American Journal of Bioethics, 19(8): 17–19. DOI: 10.1080/15265161.2019.1619880 31544641

[R36] Haklay M, Dörler D, Heigl F, Manzoni M, Hecker S and Vohland K 2021. What Is citizen science? The challenges of definition. In The Science of Citizen Science, Vohland K, Land-Zandstra A, Ceccaroni L, Rob Lemmens J, Ponti PM, Samson R and Wagenknecht K (eds.), 13–33. Cham, Switzerland: Springer. DOI: 10.1007/978-3-030-58278-4_2

[R37] Hecker S, Wicke N, Haklay M and Bonn A 2019. How does policy conceptualise citizen science? A qualitative content analysis of international policy documents. Citizen Science: Theory and Practice, 4(1): 32. DOI: 10.5334/cstp.230

[R38] Jennings P and Hussain S 2020. Do-it-yourself artificial pancreas systems: A Review of the emerging evidence and insights for healthcare professionals. Journal of Diabetes Science and Technology, 14(5): 868–877. DOI: 10.1177/1932296819894296 31847570 PMC7753866

[R39] Jwa A 2015. Early adopters of the magical thinking cap: a study on do-it-yourself (DIY) transcranial direct current stimulation (tDCS) user community. Journal of Law and the Biosciences, 2(2): 292–335. DOI: 10.1093/jlb/lsv017 27774197 PMC5034380

[R40] Kempner J and Bailey J 2019. Collective self-experimentation in patient-led research: How online health communities foster innovation. Social Science & Medicine, 238: 112366. DOI: 10.1016/j.socscimed.2019.112366 31345612

[R41] Khoruts A, Hoffmann DE and Palumbo FB. 2019. The impact of regulatory Policies on the future of fecal microbiota transplantation. The Journal of Law, Medicine & Ethics, 47(4): 482–504. DOI: 10.1177/1073110519897726 31957587

[R42] King LS. 1967. Do-it-yourself medicine. JAMA, 200(1): 23–29. DOI: 10.1001/jama.1967.03120140081012 6071850

[R43] Koebler J 2022. Anarchist collective shares instructions to make DIY abortion pills. Vice, 3 May. Available at: https://www.vice.com/en/article/qjby4b/anarchist-collective-shares-instructions-to-make-diy-abortion-pills. Accessed on September 5, 2022.

[R44] Kremer W 2014. The brave new world of DIY faecal transplant. BBC, 27 May. Available at: https://www.bbc.com/news/magazine-27503660. Accessed on August 17, 2022.

[R45] Kullenberg C and Kasperowski D 2016. What Is citizen science? – a scientometric meta-analysis. PLoS ONE, 11(1): e0147152. DOI: 10.1371/journal.pone.0147152 26766577 PMC4713078

[R46] Landrain T, Meyer M, Perez AM and Sussan R 2013. Do-it-yourself biology: challenges and promises for an open science and technology movement. Systems and Synthetic Biology, 7(3): 115–126. DOI: 10.1007/s11693-013-9116-4 24432149 PMC3740105

[R47] Lesté-Lasserre C 2020. This $1 hearing aid could treat millions with hearing loss. Science. DOI: 10.1126/science.abe9371

[R48] Lewis D 2019. History and perspective on DIY closed looping. Journal of Diabetes Science and Technology, 13(4): 790–793. DOI: 10.1177/1932296818808307 30348013 PMC6610599

[R49] Lewis D, Leibrand S and #OpenAPS Community. 2016. Real-world use of open source artificial pancreas systems. Journal of Diabetes Science and Technology, 10(6): 1411–1411. DOI: 10.1177/1932296816665635 27510442 PMC5094342

[R50] Lewis DM and Hussain S 2022. Practical guidance on open source and commercial automated insulin delivery systems: A guide for healthcare professionals supporting people with insulin-requiring diabetes. Diabetes Therapy, 1–17. DOI: 10.1007/s13300-022-01299-9 PMC9399331 35913655

[R51] Litchman ML, Lewis D, Kelly LA and Gee PM. 2019. Twitter analysis of #OpenAPS DIY artificial pancreas technology use suggests improved A1C and quality of life. Journal of Diabetes Science and Technology, 13(2): 164–170. DOI: 10.1177/1932296818795705 30198751 PMC6399802

[R52] Ma Y, Liu J, Rhodes C, Nie Y and Zhang F 2017. Ethical issues in fecal microbiota transplantation in practice. The American Journal of Bioethics, 17(5): 34–45. DOI: 10.1080/15265161.2017.1299240 28430065

[R53] Manero A, Smith P, Sparkman J, Dombrowski M, Courbin D, Kester A, Womack I and Chi A 2019. Implementation of 3D printing technology in the field of prosthetics: Past, present, and future. International Journal of Environmental Research and Public Health, 16(9): 1641. DOI: 10.3390/ijerph16091641 31083479 PMC6540178

[R54] Marty R 2019. Handbook for a Post-Roe America. New York City: Seven Stories Press.

[R55] Maschião LF, Bastos FI, Wilson E, McFarland W, Turner C, Pestana T and Veras MA. 2020. Nonprescribed sex hormone use among trans women: The complex interplay of public policies, social context, and discrimination. Transgender Health, 5(4): 205–215. DOI: 10.1089/trgh.2020.0012 33644312 PMC7906234

[R56] McClurg L 2018. Do DIY medical tests promise more than they can deliver? NPR, 28 May. Available at: https://www.npr.org/sections/health-shots/2018/05/28/614125270/do-diy-medical-tests-promise-more-than-they-can-deliver. Accessed on August 18, 2022.

[R57] Mepham N, Bouman WP, Arcelus J, Hayter M and Wylie KR. 2014. Self‐prescribing of cross‐sex hormones. The Journal of Sexual Medicine, 11(12): 2995–3001. DOI: 10.1111/jsm.12691 25213018

[R58] Metastasio A, Negri A, Martinotti G and Corazza O 2018. Transitioning bodies: The case of self-prescribing sexual hormones in gender affirmation in individuals attending psychiatric services. Brain Sciences, 8(5): 88. DOI: 10.3390/brainsci8050088 29757929 PMC5977079

[R59] Neuman S 2020. Man dies, woman hospitalized after taking form of chloroquine to prevent COVID-19. NPR, 24 March. Available at: https://www.npr.org/sections/coronavirus-live-updates/2020/03/24/820512107/man-dies-woman-hospitalized-after-taking-form-of-chloroquine-to-prevent-covid-19. Accessed on September 2, 2022.

[R60] Omer T 2016. Empowered citizen ‘health hackers’ who are not waiting. BMC Medicine, 14(1): 118. DOI: 10.1186/s12916-016-0670-y 27530970 PMC4988004

[R61] Palmer W, Greeley SAW, Letourneau-Freiberg LR and Naylor RN. 2020. Using a do-it-yourself artificial pancreas: Perspectives from patients and diabetes providers. Journal of Diabetes Science and Technology, 14(5): 860–867. DOI: 10.1177/1932296820942258 32680447 PMC7753849

[R62] Parry-Hill. 2019. e-NABLE: DIY-AT Production in a multi-stakeholder system. Thesis, Rochester Institute of Technology. Available at https://scholarworks.rit.edu/cgi/viewcontent.cgi?article=11145&context=theses. Accessed on September 3, 2022.

[R63] Richterich A 2020. When open source design is vital: critical making of DIY healthcare equipment during the COVID-19 pandemic. Health Sociology Review, 29(2): 1–10. DOI: 10.1080/14461242.2020.1784772 33411651

[R64] Roosth S 2017. Synthetic: How Life Got Made. Chicago: University of Chicago Press. DOI: 10.7208/chicago/9780226440637.001.0001

[R65] Rosenberg CE. 2007. Our Present Complaint: American Medicine, Then and Now. John Hopkins University Press.

[R66] Rotondi NK, Bauer GR, Scanlon K, Kaay M, Travers R and Travers A 2013. Nonprescribed hormone use and self-performed surgeries: “Do-it-yourself” transitions in transgender communities in Ontario, Canada. American Journal of Public Health, 103(10): 1830–7. DOI: 10.2105/AJPH.2013.301348 23948009 PMC3780733

[R67] Sanchez NF, Sanchez JP and Danoff A 2009. Health care utilization, barriers to care, and hormone usage among male-to-female transgender persons in New York City. American Journal of Public Health, 99(4): 713–719. DOI: 10.2105/AJPH.2007.132035 19150911 PMC2661470

[R68] Santora T 2020. Ignored by doctors, transgender people turn to DIY treatments. Undark, 29 June. Available at: https://undark.org/2020/06/29/transgender-diy-treatments. Accessed on September 8, 2022.

[R69] Starr P 1982. The Social Transformation Of American Medicine: The Rise Of A Sovereign Profession And The Making Of A Vast Industry. Reprint edition. New York: Basic Books.

[R70] Tomes N 2016. Remaking the American Patient: How Madison Avenue and Modern Medicine Turned Patients into Consumers. Chapel Hill, NC: University of North Carolina Press. DOI: 10.5149/northcarolina/9781469622774.001.0001

[R71] Trejo M, Canfield I, Robinson JO and Guerrini CJ. 2020. How biomedical citizen scientists define what they do: It’s all in the name. AJOB Empirical Bioethics, 12(1): 1–8. DOI: 10.1080/23294515.2020.1825139 32990526 PMC8021393

[R72] Westgarth D 2021. The alarming rise of DIY dentistry. BDJ In Practice, 34(12): 10–14. DOI: 10.1038/s41404-021-0974-2

[R73] Wexler A 2015. A pragmatic analysis of the regulation of consumer transcranial direct current stimulation (tDCS) devices in the United States. Journal of Law and the Biosciences, 2(3): 669–696. DOI: 10.1093/jlb/lsv039 27774217 PMC5034391

[R74] Wexler A 2016. The practices of do-it-yourself brain stimulation: implications for ethical considerations and regulatory proposals. Journal of Medical Ethics, 42(4): 211. DOI: 10.1136/medethics-2015-102704 26324456

[R75] Wexler A 2017a. Recurrent themes in the history of the home use of electrical stimulation: Transcranial direct current stimulation (tDCS) and the medical battery (1870–1920). Brain Stimulation, 10(2): 187–195. DOI: 10.1016/j.brs.2016.11.081 27965065

[R76] Wexler A 2017b. The social context of “do-it-yourself” brain stimulation: Neurohackers, biohackers, and lifehackers. Frontiers in Human Neuroscience, 11: 224. DOI: 10.3389/fnhum.2017.00224 28539877 PMC5423946

[R77] Wexler A 2018. Who uses direct-to-consumer brain stimulation products, and shy? A study of home users of tDCS Devices. Journal of Cognitive Enhancement, 2(1): 114–134. DOI: 10.1007/s41465-017-0062-z

[R78] Wexler A and Hamilton RH. 2017. Crowdsourced tDCS research: Feasible or fanciful? AJOB Neuroscience, 8(1): 50–53. DOI: 10.1080/21507740.2017.1288176

[R79] Wicks P, Vaughan TE, Massagli MP and Heywood J 2011. Accelerated clinical discovery using self-reported patient data collected online and a patient-matching algorithm. Nature Biotechnology, 29(5): 411–414. DOI: 10.1038/nbt.1837 21516084

[R80] Wiggins A and Wilbanks J 2019. The rise of citizen science in health and biomedical research. The American Journal of Bioethics, 19(8): 3–14. DOI: 10.1080/15265161.2019.1619859 31339831

[R81] Wurzman R, Hamilton RH, Pascual-Leone A and Fox MD. 2016. An open letter concerning do-it-yourself users of transcranial direct current stimulation. Annals of Neurology, 80(1): 1–4. DOI: 10.1002/ana.24689 27216434 PMC6050584

